# Editorial: Strengthening the Community Health Worker Practice

**DOI:** 10.3389/fpubh.2023.1254264

**Published:** 2023-08-25

**Authors:** Lily K. Lee, Julie St. John, Durrell J. Fox, E. Lee Rosenthal

**Affiliations:** ^1^KTE Strategies, LLC, Corona, CA, United States; ^2^Department of Public Health, Texas Tech University Health Sciences Center, Julia Jones Matthews School of Population and Public Health, Abilene, TX, United States; ^3^Health Services Division, JSI Research and Training Institute, Atlanta, GA, United States; ^4^Department of Medical Education, Texas Tech University Health Sciences Center El Paso, El Paso, TX, United States

**Keywords:** integration, CHWs, capacity building, organizational readiness, evaluation, certification, leadership, practice

The Editorial Team is pleased to release this second Research Topic on Community Health Workers (CHW) to bring forward trends in the field including recruitment, integration, (https://www.frontiersin.org/research-topics/15204/community-health-workers-practice-from-recruitment-to-integration) and now in this issue featuring more analysis of CHW trends in these same areas with an emphasis on: CHW capacity building; organizational and system readiness for CHW integration; championing CHW professionals and preserving the profession; CHW program evaluation and; methods and CHW certification. “Working to strengthen CHW practice” means taking time to hear voices that are often unheard: CHWs and those they serve; contributors often unknown; CHWs and allies; and even potential authors—CHWs and those in practice roles—who have begun to tell the story of their work along with the capable traditional research community of authors presented here. We looked for all of these to be represented in the two issues that comprise this Research Topic. See more about the breadth and depth of this issue in the discussion that follows.

The continuing U.S. healthcare crisis of rising healthcare costs and poor health outcomes have pushed public health and medical systems to seek solutions to address persistent gaps. Studies have revealed the deeper roots of this crisis: medical care accounts for only 20% of the variation in health outcomes for a population, while social determinants of health account for 80% (1). Community Health Workers (CHWs) who have worked for generations as trusted members of the community they serve and as trusted public health messengers, have been a workforce that has served as a focus of attention to address these deeper roots. Research, evaluation, and other reports have documented CHWs' unique ability to engage with communities and individual community members, facilitate care coordination with health and health-related providers, enhance access to community-based services, address social determinants of health, and provide health education for prevention and disease management. As a result, although CHWs are non-clinical in their scope of practice, CHWs are increasingly integrated into complex health care and managed care plan organizations. States are looking for more sustainable financing mechanisms for CHWs through Medicaid State Plan Amendments (SPAs) and 1,115 waivers, while managed care organizations and health plans are exploring creative incentives to support healthcare organizations to adopt the CHW integrated model (2). Yet, there is very little discussion around the complexities of integrating a traditionally grassroots, community-based, non- clinical workforce into complex clinical workspaces driven by licensure, hierarchy, and strict compliance policies and processes, while protecting CHWs' unique roots and community connections.

This special CHW issue provides a collection of papers addressing CHW capacity building, organizational and systems readiness, championing and preserving the profession, examining CHW program evaluation methods, CHW credentialing opportunities, and other work recognizing CHWs' contributions to improving population health and community wellness. Consistent with the spirit of, “Nothing about us without us,” the call sought out papers where CHWs served in direct roles in the projects and programs as well as in the writing as co-authors and contributors of the manuscripts.

Similarly, the editorial team invited CHWs as peer reviewers of the manuscripts ultimately featured in this issue.

## Capacity building

CHW workforce capacity building generally involves CHW core training, formal education with college-credits, continuing education covering specific topics, and other professional development opportunities. In recent years, CHWs have also advanced their careers through leadership opportunities in CHW networks and associations, coalitions, alliances, and collaboratives. To further support CHW workforce capacity, CHW programs have also explored their capacities related to financing and sustainability, recruitment and retention, program outcome evaluation and quality improvement. CHW training curricula specifically designed to meet the needs of priority populations have also emerged in recent years. Kitzie et al. present their curriculum and instruction approach to address disparities in the LGBTQIA+ communities. The 30-h LGBTQIA+ specialty training was co-developed by CHWs and researchers with expertise in LGBTQIA+ populaions and health information. The curriculum was “theater tested” and piloted with a cohort of 11 LGBTQIA+ CHWs. Their findings reveal future opportunities to train medical and nursing professionals and staff using a similar pedagogical framework. Jiménez et al.  discuss a CHW/R workforce capacity building innovation implemented during COVID-19 pandemic. A partnership between a statewide CHW professional association and an academic research team facilitated the development of community-grounded tools and resources for “rapid decision-making and knowledge sharing” to support CHWs in the field.

## Organizational and system readiness for CHW integration

Integration of CHWs into interdisciplinary teams and into leadership positions is not as intuitive as integrating clinical workforce team members, such as nurses or clinical social workers. For organizations and systems who are new to the CHW model, organizational readiness is critical. The following papers discuss approaches to training supervisors and managers, as well as lessons learned around CHWs in leadership roles. Sabo et al. share their continuous work with tribal CHWs known as Community Health Representatives (CHRs). Their paper discusses CHRs and CHR managers' involvements and perceived level of integration within health care teams and the broader public health systems addressing the social and structural determinants of health. They also discuss lessons learned from the COVID-19 pandemic responses in the tribes. Wennerstrom et al. discuss their original research that included a national cross-sectional survey of CHWs working with Medicaid managed care organizations (MCOs). Key findings from this research related to integration of CHWs into MCOS included: 85% of CHWs made referrals; 75% conducted social screenings; 54% assisted with care planning; 52% conducted health screenings; and 49% participated in case reviews (49.3%). Wennerstrom et al. suggest that CHWs' roles in MCOs focus on supporting clinical care and making referrals for social issues and not on community-level concerns. The authors recommend that MCOs ensure that CHWs: have professional freedom to develop community- based solutions; receive equitable compensation; and have promotion opportunities. Jeyakumar et al.'s original research study explored the current functioning and sustainability of Aboriginal Health Workers (AHWs)—core primary healthcare (PHC) providers— of First Nations peoples in New South Whales (NSW) PHC organizations. Results identified five categories of change required to ensure AHW sustainability and retention: community connection, recognition, value, support, and an inclusive health system—with both service and system level factors influencing each change category. The authors conclude that ensuring sustainability of the AHW workforce will require a system-wide paradigm shift that includes holistic health approaches and suggest the need for future studies co-designed with ACCHOs (Aboriginal Community Controlled Health Organizations) to help inform this change.

## Championing CHW professionals and preserving the profession

In addition to building CHW capacity and organizational readiness, organizations successful in CHW integration share some common strategies. Success begins with the right recruitment—individuals from the communities they serve with lived experiences who demonstrate key CHW qualities and attributes (3). Several papers discuss identifying champions for the CHW workforce within the organization who understand CHW core competencies and scope of practice, and advocate for the preservation of the CHW profession, creating career pathways, safety and boundaries, and bridging gaps in understanding CHW integrated practice dynamics. In their opinion paper, Masquillier and Cosaert discuss some arguments of CHW integration in health systems, such as an innovation or just an emergency response through the COVID-19 pandemic. In Belgium, the pandemic served as a catalyst that revealed various gaps and disparities in the system, including shortages and unmet needs of socio- economically vulnerable communities. The authors elevate the need to build the CHWs' role in supporting “equitable and accessible healthcare for all, by looking beyond emergency responses.” Strengthening the CHW workforce involves formulating a long-term vision and ensuring sustainable funding, both of which were “set in motion by the COVID-19 pandemic.” In their original mixed methods research study, Ajisegiri et al. explored CHW roles and practices related to the delivery of non-communicable disease (NCD) services at primary health care (PHC) facilities in four Nigerian states; traditional CHW work in Nigeria has focused on infectious diseases and maternal and child health services. Study findings demonstrated that CHWs frequently delivered services beyond the scope of practice stipulated in the Nigerian National Standing Orders for CHWs; the need to serve the community primarily motivated these informal task-shifting practices. The authors assert that provision of services related to NCDs both partially support health system functions and address unmet needs but could also lead to variable care quality and safety. The authors recommend ways to mitigate potential adverse impacts and to strengthen CHW roles in the health system, including: a stronger enabling policy environment to support NCD task-sharing; investment in continuous CHW capacity building; improved guidelines for implementation at the point of care, and improved coordination processes between PHC and higher-level facilities. In their original research, Smithwick et al. conducted a mixed-method study on the limited professional and career building pathways for CHWs contributing to lower wages, lack of career advancement, turnover, attrition, and workforce instability. Study findings stressed the importance of retaining skilled and experienced CHWs and educating health professions about CHWs' critical roles, which will decrease attrition, enhance professional growth, and improve program quality. Findings also suggest that higher wages, valuing lived experience over formal education, and participation in additional training opportunities should constitute the primary factors considered for career advancement.

## CHW program evaluation and methods

As more CHWs become integrated into public health and healthcare systems, assessing the continuous impact CHWs have on population health management outcomes is important. These assessments are often criticized for lacking proper methodologies or participants' protection, especially those in more vulnerable populations. Killough et al. present a brief research report on CHW's unique perspectives of frontline CHWs who identified actionable barriers and facilitators that may impact representation of diverse groups in health research. As trusted members of the community, CHWs recognize their roles as facilitators in ensuring their community receive resources and benefit from being involved in research; as gatekeepers, CHWs take on the role of protectors so their community doesn't experience further trauma when engaging in research. Bush et al. present original research focused on the evaluation of a CHW-led self-management blood pressure (SMBP) program, which strove to improve hypertension management through raising awareness, education, navigation, advocacy, and resource assistance. Outcome measures indicate this CHW-led intervention improved management of hypertension through education on lifestyle changes (including creating lifelong healthy habits, coping skills, stress management, self-care, and accountability) impacting overall health and quality of life.

## CHW certification

With any profession, validation of their competencies through some process of certification, licensure or registration is required. These requirements are more stringent for those working in healthcare settings. For the CHW workforce, more states have adopted or are examining CHW certification, which are often tied to requirements for billing, which in turn have direct implications on sustainability. Nielsen et al. share their lessons learned through an evaluation of the impact of the CHW certification in Massachusetts. Through a CHW survey pre-certification in 2016 and post- certification in 2021, they report on the 5-year efforts to a statewide CHW certification, as well as the impact. Their findings also revealed important gaps in the CHW research that are much needed to build a broader bank of knowledge in this area.

## Conclusion

This Research Topic of eleven articles highlighted the following key areas needing further examination in order to continue to strengthen the community health worker practice. First, CHW programs, employers, and supervisors must acknowledge and commit to involving CHWs in all aspects of programs, leadership, and decision-making, aligning with “Nothing about us without us.” Further research is needed on how to engage CHWs equitably. Second, the CHW practice has moved from acknowledging the essential role of CHWs to establishing and implementing a vision for CHW integration. The COVID-19 pandemic exposed many disparities and layers of population health management where CHWs can play impactful roles, especially in healthcare settings. Future studies can focus on actual integration practices of this vital workforce into complex systems and the impact value of CHW-integrated practices and CHW roles. Finally, funding support for CHW sustainability is still a major issue. More studies are needed on developing funding models to sustain the workforce. This Research Topic may not offer a complete picture but rather insights to building the CHW workforce and elements for strengthening the practice. We hope these articles shed new knowledge to inform and guide your practice. We also hope to build on efforts in these two issues that make up this Research Topic to lift up unheard community members, CHWs, and other voices in frontline practice settings to help us deepen our understanding of the field. We also expect to build on the tradition of CHWs playing roles as Peer Reviewers in future editorial endeavors to ensure authentic representation of voices—and most importantly, the accurate representation of CHW practice in published work about the CHW field.

## Author contributions

LL: Conceptualization, Methodology, Writing—original draft, Writing—review and editing. JJ: Conceptualization, Methodology, Writing—original draft, Writing—review and editing. DF: Conceptualization, Methodology, Writing—review and editing. ER: Conceptualization, Methodology, Writing—review and editing.
